# *N*-(7-Eth­oxy-1*H*-indazol-4-yl)-4-methyl­benzene­sulfonamide

**DOI:** 10.1107/S1600536811016576

**Published:** 2011-05-07

**Authors:** Najat Abbassi, El Mostapha Rakib, Hafid Zouihri

**Affiliations:** a Laboratoire de Chimie Organique et Analytique, Université Sultan Moulay Slimane, Faculté des Sciences et Techniques, Béni-Mellal, BP 523, Morocco; b Laboratoires de Diffraction des Rayons X, Centre Nationale pour la Recherche Scientifique et Technique, Rabat, Morocco

## Abstract

The mol­ecule of the title heterocyclic compound, C_16_H_17_N_3_O_3_S, is bent at the S atom with an C—SO_2_—NH—C torsion angle of 80.17 (8)°. The phenyl substituent at the S atom is rotated out of the plane of the 1*H*-indazole ring [inter­planar angle = 46.24 (8)°]. In the crystal, inter­molecular N—H⋯N and N—H⋯O hydrogen bonds build up a ribbon developing parallel to the *b*-axis direction. C—H⋯O hydrogen bonds link these ribbons, forming a layer parallel to the *bc* plane.

## Related literature

For related structures, see: Shakuntala *et al.* (2011*a* ▶,*b* ▶); Khan *et al.* (2010 ▶); Gowda *et al.* (2010 ▶). For the biological activity of similar sulfonamides, see: Soledade *et al.* (2006 ▶); Lee & Lee, (2002 ▶).
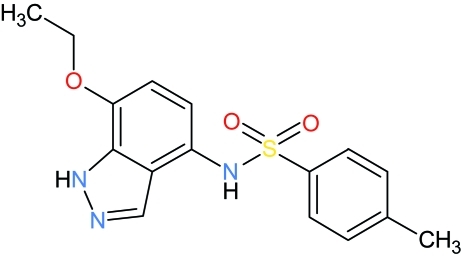


## Experimental

### 

#### Crystal data

C_16_H_17_N_3_O_3_S
*M* *_r_* = 331.39
Monoclinic, 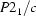

*a* = 16.2579 (4) Å
*b* = 5.0291 (1) Å
*c* = 20.4551 (5) Å
β = 97.269 (1)°
*V* = 1659.02 (7) Å^3^
*Z* = 4
Mo *K*α radiation
μ = 0.21 mm^−1^
*T* = 296 K
0.23 × 0.20 × 0.14 mm

#### Data collection

Bruker APEXII CCD detector diffractometer
31243 measured reflections
6745 independent reflections
5062 reflections with *I* > 2σ(*I*)
*R*_int_ = 0.025

#### Refinement

*R*[*F* ^2^ > 2σ(*F* ^2^)] = 0.053
*wR*(*F* ^2^) = 0.158
*S* = 1.07
6745 reflections
210 parameters
H-atom parameters constrained
Δρ_max_ = 0.42 e Å^−3^
Δρ_min_ = −0.25 e Å^−3^

### 

Data collection: *APEX2* (Bruker, 2005 ▶); cell refinement: *SAINT* (Bruker, 2005 ▶); data reduction: *SAINT*; program(s) used to solve structure: *SHELXS97* (Sheldrick, 2008 ▶); program(s) used to refine structure: *SHELXL97* (Sheldrick, 2008 ▶); molecular graphics: *PLATON* (Spek, 2009 ▶); software used to prepare material for publication: *publCIF* (Westrip, 2010 ▶).

## Supplementary Material

Crystal structure: contains datablocks I, global. DOI: 10.1107/S1600536811016576/dn2681sup1.cif

Structure factors: contains datablocks I. DOI: 10.1107/S1600536811016576/dn2681Isup2.hkl

Supplementary material file. DOI: 10.1107/S1600536811016576/dn2681Isup3.mol

Supplementary material file. DOI: 10.1107/S1600536811016576/dn2681Isup4.cml

Additional supplementary materials:  crystallographic information; 3D view; checkCIF report

## Figures and Tables

**Table 1:** Hydrogen-bond geometry (Å, °)

*D*—H⋯*A*	*D*—H	H⋯*A*	*D*⋯*A*	*D*—H⋯*A*
C14—H14*B*⋯O1^i^	0.97	2.54	3.373 (3)	144
N1—H14⋯O2^ii^	0.85	2.07	2.9159 (15)	172
N2—H2*A*⋯N3^iii^	0.86	2.21	2.8974 (15)	136

Symmetry codes: (i) 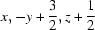; (ii) 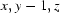; (iii) 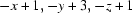.

**Fig. 1.**
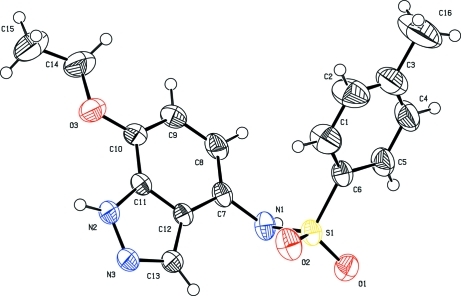
Molecular view of the title compound showing the atom-labeling scheme. Displacement ellipsoids are drawn at the 50% probability level. H atoms are represented as small spheres of arbitrary radii.

**Fig. 2.**
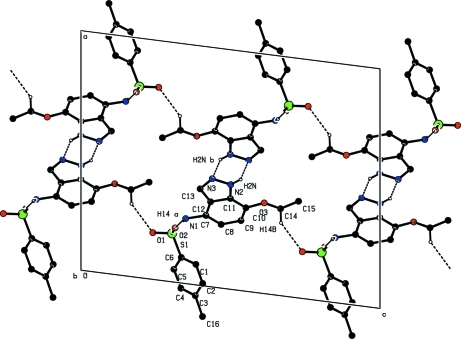
Partial packing view showing the chain formed by C—H···O, N—H···O and N—H···N hydrogen bondings. H atoms not involved in hydrogen bonds have been omitted for clarity.

## supplementary crystallographic information

### Comment

Similar sulfonamides have been studied in various previously works [Shakuntala *et al.* 2011*a* and 2011*b*; Khan *et al.* 2010; Gowda *et al.* 2010] and have proved important functionalities for biological and anti-hypertensive activities [Soledade *et al.*, 2006; Lee & Lee, 2002].

In the title compound, C_16_H_17_N_3_O_3_S, the molecule is bent at the S atom with an C—SO_2_—NH—C torsion angle of 80.17 (8)° (Fig. 1). In the crystal structure, intermolecular N—H···N and N—H···O hydrogen bonds build up a ribbon developping parallel to the b direction and the C—H···O link these reibons to form a two D layer parallel to the *bc* plane (Fig. 2, Table 1).

The S atom has a distorted tetrahedral geometry [maximum deviation: O—S—O = 119.87 (9)°]. The phenyl substituent at S1 atom is rotated out of the plane of the 1*H*-indazol ring (the interplanar angles is 46.24 (8)°).

### Experimental

A mixture of 4-nitroindazole (1.22 mmol) and anhydrous SnCl_2_ (1.1 g, 6.1 mmol) in 25 mL of absolute ethanol was heated at 60 °C for 2 h. After reduction, the starting material disappeared, and the solution was allowed to cool down. The pH was made slightly basic (pH 7–8) by addition of 5% aqueous potassium bicarbonate before extraction with ethyl acetate. The organic phase was washed with brine and dried over magnesium sulfate. The solvent was removed to afford the amine, which was immediately dissolved in pyridine (5 ml) and then reacted with 4-methylbenzenesulfonyl chloride (0.26 g, 1.25 mmol) at room temperature for 24 h. After the reaction mixture was concentrated in vacuo, the resulting residue was purified by flash chromatography (eluted with Ethyl acetate: Hexane 1:9).

Yield: 45%; mp: 443–445 °K; IR (KBr, cm^-1^): 3340, 3235 (NH), 1595 (CN), 1335, 1160 (SO_2_); ^1^H NMR (DMSO-d_6_): 2.27 (s, 3H, CH3), 6.92 (dd, 1H, J=2.1 Hz and 6.1 Hz), 7.16 (d, 2H, J=6.2 Hz), 7.28 (d, 2H, J=8.3 Hz), 7.68 (d, 2H, J=8.3 Hz), 8.22 (s, 1H),10.51 (s, 1H, NH), 13.05 (s, 1H, NH); ^13^C NMR (DMSO-d_6_): 21.3 (CH3), 106.7,111.0, 126.9, 127.2, 130.1, 132.4 (6 CH), 117.3, 130.5, 137.3, 141.4, 143.7 (5 C); MS m/*z*=288 [*M*+1].

### Refinement

The H atoms bound to C were treated as riding with their parent atoms [C—H distances are 0.93Å for CH groups and 0.96Å for CH2 with *U*_iso_(H) = 1.2 *U*_eq_(C), and 0.97 Å for CH3 groups with *U*_iso_(H) = 1.5 *U*_eq_(C). The N2—H20 H atoms were treated as riding with *U*_iso_(H) = 1.2 *U*_eq_(N), and the N1—H10 H atoms were refined with restraints (d_N–H_ = 0.88 (2) Å) and then were treated as riding in the last cycles of refinement.

### Crystal data

**Table:**

C_16_H_17_N_3_O_3_S	*F*(000) = 696
*M**_r_* = 331.39	*D*_x_ = 1.327 Mg m^−^^3^
Monoclinic, *P*2_1_/*c*	Melting point: 445 K
Hall symbol: -P 2ybc	Mo *K*α radiation, λ = 0.71073 Å
*a* = 16.2579 (4) Å	Cell parameters from 347 reflections
*b* = 5.0291 (1) Å	θ = 2.7–27.2°
*c* = 20.4551 (5) Å	µ = 0.21 mm^−^^1^
β = 97.269 (1)°	*T* = 296 K
*V* = 1659.02 (7) Å^3^	Prism, yellow
*Z* = 4	0.23 × 0.20 × 0.14 mm

### Data collection

**Table:**

Bruker APEXII CCD detector diffractometer	5062 reflections with *I* > 2σ(*I*)
Radiation source: fine-focus sealed tube	*R*_int_ = 0.025
graphite	θ_max_ = 34.0°, θ_min_ = 1.3°
ω and φ scans	*h* = −25→25
31243 measured reflections	*k* = −7→7
6745 independent reflections	*l* = −32→31

### Refinement

**Table:**

Refinement on *F*^2^	Primary atom site location: structure-invariant direct methods
Least-squares matrix: full	Secondary atom site location: difference Fourier map
*R*[*F*^2^ > 2σ(*F*^2^)] = 0.053	Hydrogen site location: inferred from neighbouring sites
*wR*(*F*^2^) = 0.158	H-atom parameters constrained
*S* = 1.07	*w* = 1/[σ^2^(*F*_o_^2^) + (0.0799*P*)^2^ + 0.3561*P*] where *P* = (*F*_o_^2^ + 2*F*_c_^2^)/3
6745 reflections	(Δ/σ)_max_ = 0.011
210 parameters	Δρ_max_ = 0.42 e Å^−^^3^
0 restraints	Δρ_min_ = −0.25 e Å^−^^3^

### Special details

**Table:**

Geometry. All esds (except the esd in the dihedral angle between two l.s. planes) are estimated using the full covariance matrix. The cell esds are taken into account individually in the estimation of esds in distances, angles and torsion angles; correlations between esds in cell parameters are only used when they are defined by crystal symmetry. An approximate (isotropic) treatment of cell esds is used for estimating esds involving l.s. planes.
Refinement. Refinement of *F*^2^ against ALL reflections. The weighted *R*-factor *wR* and goodness of fit *S* are based on *F*^2^, conventional *R*-factors *R* are based on *F*, with *F* set to zero for negative *F*^2^. The threshold expression of *F*^2^ > σ(*F*^2^) is used only for calculating *R*-factors(gt) *etc*. and is not relevant to the choice of reflections for refinement. *R*-factors based on *F*^2^ are statistically about twice as large as those based on *F*, and *R*- factors based on ALL data will be even larger.

### Fractional atomic coordinates and isotropic or equivalent isotropic displacement parameters (Å^2^)

**Table:**

	*x*	*y*	*z*	*U*_iso_*/*U*_eq_	
S1	0.202461 (19)	0.91282 (6)	0.304901 (17)	0.03746 (10)	
N1	0.27119 (7)	0.7334 (2)	0.35009 (6)	0.0391 (2)	
H14	0.2635	0.5696	0.3412	0.047*	
N2	0.43721 (7)	1.2628 (2)	0.50079 (6)	0.0384 (2)	
H2A	0.4616	1.3343	0.5362	0.046*	
N3	0.45193 (7)	1.3298 (3)	0.43916 (6)	0.0416 (3)	
O1	0.19501 (7)	0.8007 (3)	0.24030 (5)	0.0519 (3)	
O2	0.22674 (7)	1.1855 (2)	0.31483 (6)	0.0524 (3)	
O3	0.37858 (8)	1.0205 (3)	0.61280 (6)	0.0578 (3)	
C1	0.08572 (13)	1.0406 (4)	0.38431 (11)	0.0631 (5)	
H1	0.1221	1.1725	0.4018	0.076*	
C2	0.00971 (15)	1.0085 (5)	0.40702 (13)	0.0794 (7)	
H2	−0.0048	1.1212	0.4398	0.095*	
C3	−0.04508 (12)	0.8132 (5)	0.38215 (12)	0.0712 (6)	
C4	−0.02207 (11)	0.6462 (5)	0.33422 (11)	0.0651 (5)	
H4	−0.0581	0.5121	0.3174	0.078*	
C5	0.05334 (10)	0.6742 (4)	0.31072 (9)	0.0515 (4)	
H5	0.0681	0.5593	0.2785	0.062*	
C6	0.10683 (8)	0.8748 (3)	0.33547 (7)	0.0385 (3)	
C7	0.29527 (7)	0.8035 (2)	0.41776 (7)	0.0346 (2)	
C8	0.26575 (9)	0.6722 (3)	0.46859 (8)	0.0451 (3)	
H8	0.2269	0.5376	0.4590	0.054*	
C9	0.29232 (10)	0.7346 (3)	0.53502 (8)	0.0501 (4)	
H9	0.2716	0.6385	0.5682	0.060*	
C10	0.34852 (9)	0.9356 (3)	0.55166 (7)	0.0415 (3)	
C11	0.37907 (7)	1.0689 (2)	0.49948 (6)	0.0338 (2)	
C12	0.35467 (7)	1.0062 (2)	0.43349 (6)	0.0323 (2)	
C13	0.40308 (8)	1.1763 (3)	0.39843 (7)	0.0381 (3)	
H13	0.4008	1.1797	0.3528	0.046*	
C14	0.36325 (16)	0.8578 (6)	0.66736 (10)	0.0814 (7)	
H14A	0.3858	0.6811	0.6630	0.098*	
H14B	0.3041	0.8422	0.6691	0.098*	
C15	0.4038 (2)	0.9868 (8)	0.72770 (11)	0.1024 (9)	
H15A	0.4610	1.0202	0.7232	0.154*	
H15B	0.4005	0.8722	0.7648	0.154*	
H15C	0.3765	1.1520	0.7344	0.154*	
C16	−0.12866 (17)	0.7785 (10)	0.40632 (19)	0.1314 (14)	
H16A	−0.1231	0.6624	0.4439	0.197*	
H16B	−0.1671	0.7026	0.3718	0.197*	
H16C	−0.1489	0.9484	0.4186	0.197*	

### Atomic displacement parameters (Å^2^)

**Table:**

	*U*^11^	*U*^22^	*U*^33^	*U*^12^	*U*^13^	*U*^23^
S1	0.03566 (15)	0.03045 (15)	0.04463 (19)	−0.00315 (11)	−0.00135 (12)	−0.00309 (12)
N1	0.0377 (5)	0.0267 (4)	0.0501 (6)	−0.0007 (4)	−0.0058 (4)	−0.0085 (4)
N2	0.0355 (5)	0.0403 (5)	0.0385 (5)	−0.0137 (4)	0.0015 (4)	−0.0025 (4)
N3	0.0373 (5)	0.0444 (6)	0.0427 (6)	−0.0155 (5)	0.0038 (4)	0.0018 (5)
O1	0.0506 (6)	0.0608 (7)	0.0429 (6)	−0.0006 (5)	0.0011 (4)	−0.0066 (5)
O2	0.0501 (6)	0.0289 (5)	0.0755 (8)	−0.0068 (4)	−0.0021 (5)	0.0035 (5)
O3	0.0651 (7)	0.0711 (8)	0.0380 (5)	−0.0210 (6)	0.0100 (5)	−0.0019 (5)
C1	0.0635 (10)	0.0507 (9)	0.0779 (13)	−0.0091 (8)	0.0196 (9)	−0.0217 (9)
C2	0.0760 (14)	0.0735 (13)	0.0963 (17)	−0.0007 (12)	0.0407 (13)	−0.0193 (13)
C3	0.0481 (9)	0.0836 (14)	0.0844 (14)	−0.0007 (9)	0.0185 (9)	0.0131 (12)
C4	0.0438 (8)	0.0742 (12)	0.0748 (12)	−0.0200 (8)	−0.0020 (8)	0.0052 (10)
C5	0.0452 (7)	0.0516 (8)	0.0561 (9)	−0.0130 (6)	−0.0006 (6)	−0.0089 (7)
C6	0.0352 (5)	0.0330 (6)	0.0457 (7)	−0.0009 (4)	−0.0012 (5)	−0.0021 (5)
C7	0.0296 (5)	0.0273 (5)	0.0457 (7)	−0.0034 (4)	−0.0001 (4)	−0.0027 (5)
C8	0.0384 (6)	0.0372 (6)	0.0587 (9)	−0.0146 (5)	0.0027 (6)	0.0028 (6)
C9	0.0454 (7)	0.0533 (8)	0.0528 (8)	−0.0178 (6)	0.0110 (6)	0.0081 (7)
C10	0.0382 (6)	0.0464 (7)	0.0404 (6)	−0.0082 (5)	0.0076 (5)	0.0017 (5)
C11	0.0288 (5)	0.0326 (5)	0.0400 (6)	−0.0053 (4)	0.0041 (4)	−0.0007 (5)
C12	0.0278 (4)	0.0299 (5)	0.0387 (6)	−0.0046 (4)	0.0019 (4)	−0.0013 (4)
C13	0.0360 (5)	0.0406 (6)	0.0375 (6)	−0.0094 (5)	0.0040 (4)	0.0004 (5)
C14	0.0892 (15)	0.1092 (19)	0.0474 (10)	−0.0299 (14)	0.0152 (10)	0.0098 (11)
C15	0.118 (2)	0.146 (3)	0.0443 (11)	−0.024 (2)	0.0118 (12)	−0.0018 (14)
C16	0.0683 (16)	0.171 (4)	0.165 (3)	−0.012 (2)	0.058 (2)	0.001 (3)

### Geometric parameters (Å, °)

**Table:**

S1—O1	1.4277 (11)	C5—C6	1.3854 (19)
S1—O2	1.4345 (11)	C5—H5	0.9300
S1—N1	1.6296 (11)	C7—C8	1.369 (2)
S1—C6	1.7581 (14)	C7—C12	1.4133 (16)
N1—C7	1.4340 (17)	C8—C9	1.408 (2)
N1—H14	0.8492	C8—H8	0.9300
N2—N3	1.3550 (16)	C9—C10	1.376 (2)
N2—C11	1.3561 (15)	C9—H9	0.9300
N2—H2A	0.8600	C10—C11	1.4032 (18)
N3—C13	1.3241 (17)	C11—C12	1.3939 (17)
O3—C10	1.3529 (18)	C12—C13	1.4170 (17)
O3—C14	1.431 (2)	C13—H13	0.9300
C1—C6	1.378 (2)	C14—C15	1.474 (3)
C1—C2	1.384 (3)	C14—H14A	0.9700
C1—H1	0.9300	C14—H14B	0.9700
C2—C3	1.379 (3)	C15—H15A	0.9600
C2—H2	0.9300	C15—H15B	0.9600
C3—C4	1.378 (3)	C15—H15C	0.9600
C3—C16	1.514 (3)	C16—H16A	0.9600
C4—C5	1.379 (2)	C16—H16B	0.9600
C4—H4	0.9300	C16—H16C	0.9600
			
O1—S1—O2	119.88 (8)	C7—C8—H8	118.9
O1—S1—N1	106.15 (7)	C9—C8—H8	118.9
O2—S1—N1	107.00 (6)	C10—C9—C8	121.01 (13)
O1—S1—C6	108.12 (7)	C10—C9—H9	119.5
O2—S1—C6	107.07 (7)	C8—C9—H9	119.5
N1—S1—C6	108.17 (7)	O3—C10—C9	127.66 (14)
C7—N1—S1	119.69 (9)	O3—C10—C11	115.56 (12)
C7—N1—H14	117.4	C9—C10—C11	116.78 (13)
S1—N1—H14	110.1	N2—C11—C12	107.09 (11)
N3—N2—C11	111.41 (10)	N2—C11—C10	129.89 (12)
N3—N2—H2A	124.3	C12—C11—C10	122.95 (11)
C11—N2—H2A	124.3	C11—C12—C7	119.09 (11)
C13—N3—N2	106.15 (10)	C11—C12—C13	104.21 (10)
C10—O3—C14	117.55 (15)	C7—C12—C13	136.68 (12)
C6—C1—C2	119.19 (18)	N3—C13—C12	111.14 (12)
C6—C1—H1	120.4	N3—C13—H13	124.4
C2—C1—H1	120.4	C12—C13—H13	124.4
C3—C2—C1	121.5 (2)	O3—C14—C15	107.3 (2)
C3—C2—H2	119.3	O3—C14—H14A	110.3
C1—C2—H2	119.3	C15—C14—H14A	110.3
C4—C3—C2	118.40 (17)	O3—C14—H14B	110.3
C4—C3—C16	119.9 (2)	C15—C14—H14B	110.3
C2—C3—C16	121.7 (3)	H14A—C14—H14B	108.5
C3—C4—C5	121.26 (18)	C14—C15—H15A	109.5
C3—C4—H4	119.4	C14—C15—H15B	109.5
C5—C4—H4	119.4	H15A—C15—H15B	109.5
C4—C5—C6	119.43 (17)	C14—C15—H15C	109.5
C4—C5—H5	120.3	H15A—C15—H15C	109.5
C6—C5—H5	120.3	H15B—C15—H15C	109.5
C1—C6—C5	120.23 (15)	C3—C16—H16A	109.5
C1—C6—S1	120.31 (12)	C3—C16—H16B	109.5
C5—C6—S1	119.45 (12)	H16A—C16—H16B	109.5
C8—C7—C12	118.04 (12)	C3—C16—H16C	109.5
C8—C7—N1	122.39 (11)	H16A—C16—H16C	109.5
C12—C7—N1	119.49 (11)	H16B—C16—H16C	109.5
C7—C8—C9	122.11 (12)		

### Hydrogen-bond geometry (Å, °)

**Table:**

*D*—H···*A*	*D*—H	H···*A*	*D*···*A*	*D*—H···*A*
C14—H14B···O1^i^	0.97	2.54	3.373 (3)	144
N1—H14···O2^ii^	0.85	2.07	2.9159 (15)	172
N2—H2A···N3^iii^	0.86	2.21	2.8974 (15)	136

Symmetry codes: (i) *x*, −*y*+3/2, *z*+1/2; (ii) *x*, *y*−1, *z*; (iii) −*x*+1, −*y*+3, −*z*+1.

## References

[bb1] Bruker (2005). *APEX2* and *SAINT* Bruker AXS Inc., Madison, Wisconsin, USA.

[bb2] Gowda, B. T., Foro, S., Nirmala, P. G. & Fuess, H. (2010). *Acta Cryst.* E**66**, o1702. 10.1107/S1600536810022968 PMC3006774 21587922

[bb3] Khan, I. U., Ahmad, W., Arshad, M. N., Sharif, S. & Ahmed, J. (2010). *Acta Cryst.* E**66**, o2507. 10.1107/S1600536810035105 PMC2983116 21587503

[bb4] Lee, J. S. & Lee, C. H. (2002). *Bull. Korean Chem. Soc.* **23**, 167–169.

[bb5] Shakuntala, K., Foro, S. & Gowda, B. T. (2011*a*). *Acta Cryst.* E**67**, o104. 10.1107/S1600536810051305 PMC3050347 21522617

[bb6] Shakuntala, K., Foro, S. & Gowda, B. T. (2011*b*). *Acta Cryst.* E**67**, o142. 10.1107/S1600536810051792 PMC3050268 21522651

[bb7] Sheldrick, G. M. (2008). *Acta Cryst.* A**64**, 112–122. 10.1107/S0108767307043930 18156677

[bb8] Soledade, M., Pedras, C. & Jha, M. (2006). *Bioorg. Med. Chem.* **14**, 4958–4979. 10.1016/j.bmc.2006.03.014 16616505

[bb9] Spek, A. L. (2009). *Acta Cryst.* D**65**, 148–155. 10.1107/S090744490804362X PMC2631630 19171970

[bb10] Westrip, S. P. (2010). *J. Appl. Cryst.* **43**, 920–925.

